# Effectiveness of a computer assisted learning (CAL) package to raise awareness of autism

**DOI:** 10.1186/1472-6920-9-12

**Published:** 2009-02-26

**Authors:** Jariya Chuthapisith, Benedict diMambro, Gillian Doody

**Affiliations:** 1 Department of Paediatrics, Faculty of Medicine Ramathibodi Hospital, Mahidol University, Bangkok, Thailand; 2 Division of Psychiatry, University of Nottingham, Nottingham, UK

## Abstract

**Background:**

Promoting awareness of autism in populations who work with children may result in an earlier diagnosis of the condition. In this study, a computer assisted learning (CAL) package, containing educationally appropriate knowledge about autism was developed; and the effectiveness of this CAL package was evaluated.

**Methods:**

The CAL package was developed using computer software, "Xerte" and "Flash Macromedia". The effectiveness of the CAL package was evaluated in 32 childcare students in the UK, who were randomised to watch the CAL package or to read the information leaflet containing the same information (n = 16 in each group). Retention performance, level of enjoyment, and level of confidence to identify a child with autism, after the interventions, were evaluated. The data obtained from two studied groups was analysed using unpaired Student's t-test, 95% confidence interval, and effect size.

**Results:**

Students who watched the CAL package had superior retention performance percentage scores (p = 0.02, 95% CI = 0.83–12.19, effect size = 0.8) and level of enjoyment (p = 0.04, 95% CI = 0.03–2.75, effect size = 0.7) compared with students who read the information leaflet. However, there was no significant difference in level of confidence to identify a child with autism (p = 0.39, 95% CI = -1.80–0.72, effect size = -0.3).

**Conclusion:**

The CAL package developed was an effective method of educating people who work with children about autism.

## Background

Autism is a common neurodevelopmental disorder in children. Clinical features of autism consist of three main impairments, known as the "triad of impairment": impairment in social interaction, impairment in communication and impairment in imagination. Recent research documented that prevalence for all autistic spectrum disorders (ASD) varied from 52 to 67 per 10,000, depending on geography and research methodology [1-4]. Although, there are many standard diagnostic instruments utilised to help to diagnose autism [5-7], the diagnosis of autism remains difficult, especially before the age of 3 [8]. Clinical manifestations are sometimes less obvious and not compatible with the diagnostic instruments. Consequently, the diagnosis of autism may be delayed and that may affect the outcome of children with autism. The early detection of autism can lead to early interventions which may well improve social and language development [9-15].

Although, autism is a common disorder in children, healthcare professionals and carers who work with children are all too often unaware of the details of this disorder. There exists a wide range of misconceptions about autism regarding social/emotional, cognitive, and general features among various healthcare disciplines (including paediatrics, clinical psychology, school psychology and speech/language therapy) [16]. Even medical students, parents and teachers of children with autism have demonstrated spurious knowledge about autism [17,18]. These potentially have a deleterious effect on children and families. Therefore, in order to help services meet the needs of children with autism, it is crucial for persons who work with children to be fully aware of correct information about autism. This will allow children to be referred promptly to paediatrician or specialist for an early, appropriate, and consistent interventions at younger ages, thus, potentially improving the long-term outcomes.

During the last three decades, as a consequence of advancements in technology, a number of educational multimedia materials have been developed and used in medical teaching. One of those educational multimedia materials is computer assisted learning (CAL). The benefit of CAL package in teaching medical related lesson is clearly demonstrated in some areas of medical education, particularly in clinical simulation, problem-based curricular, teaching basic anatomy and supplementing tutorials [19-23]. To the author's knowledge, there has been no published study examining the use of CAL package in autism. The aim of the study is, therefore, to evaluate the effectiveness (in terms of providing essential information about autism, users' level of enjoyment and users' level of confidence to identify a child with autism) of the CAL package, comparing with the information leaflet.

## Methods

### Participants and Procedures

The study was ethically approved by the University of Nottingham Medical School Research Ethics Committee. Thirty-two students undertaking a Childcare and Early Years course from the New College Nottingham, UK were invited into the study. Students who complete this vocational course will receive a Diploma qualification and become a qualified nursery nurse. The curriculum includes social and developmental aspects of young children, health care, community care, and interactions with parents. Abnormal development and autism are not included in the curriculum [24].

All participants were asked to read the information sheets and informed consent was obtained before commencing the study. Participants were randomised by random numbers into 2 groups: the CAL package watching and the leaflet reading groups.

Both groups were allowed to watch the CAL package or to read the information leaflet once for 15 minutes. After the intervention, retention performance (ability to remember and recall the information in the CAL package and the leaflet), level of enjoyment, and level of confidence were evaluated by the questionnaire and the visual analogue scales.

### Measurements

#### CAL package

The CAL package was developed with the "Xerte" and the "Flash Macromedia" programmes. Narration was recorded using the Audicity programme. All information presented in the CAL package was adapted from the National Autism Plan for Children (NAPC) [25] and National Autistic Society (NAS), UK [26]. The detail of the information presented was based on the depth of detail included in other subjects within the nursery nurse curriculum. There were 5 main sections in the CAL package: general knowledge, diagnosis, aetiology, co-morbidity and treatment. The CAL package lasted for12 minutes. All information appeared in the CAL package (as well as in the information leaflet) was approved by two consultants in Clinical Psychiatry.

#### Information leaflet

The information leaflet was derived from the same details as in the CAL package and was presented on two pages A4 sheet. The information was presented in the same sequence as in the CAL package. Three black and white cartoon images derived from the CAL package were also included in the leaflet.

#### Questionnaire

The questionnaire was developed under the supervision of two consultant psychiatrists. The questionnaire consisted of three main parts. The first part covered demographic details of the participants (e.g. age, level of education). The second part assessed retention performance using 24 True-False questions. The questions were categorised into 5 sections: general knowledge, diagnosis, aetiology, co-morbidity, and treatment. The questions allowed the participants to answer "true", "false" or "not sure". Each question was scored 1 if the answer was correct. On the other hand, it was scored 0 if the answer was incorrect or the participants answered "not sure" to that question. Therefore, the maximum score was 24 and the lowest score was 0. In the third part of the questionnaire, two visual analogue scales employed to evaluate level of enjoyment and level of confidence to identify a child as autism. Both scales were 100 millimetres long and the participants were asked to mark an "X" on the line to indicate level of enjoyment and level of confidence to identify a child as autism. The level of enjoyment and level of confidence ranged from "not at all" on the left end to "extremely" on the right end. They were scored by measuring the length of the line in millimeters from the left end to the mark "X". Therefore, the highest level of enjoyment and confidence was 100 and the lowest was 0. Examples of the questions in the second part of the questionnaire were included in Appendix 1.

### Analyses

All analyses were performed using SPSS 13.0 software (SPSS Inc, Chicago, IL). Descriptive analyses were undertaken for the demographic information between the groups. The Shapiro-Wilks W test was used to test normality of distribution. Unpaired Student's t-tests and 95% Confidence Interval (95% CI) were utilised to compare the post-intervention retention performance, level of enjoyment, and level of confidence to identify a child as autism between groups. Probability (P) values of or less than 0.05 were considered statistical significance. Effect size was calculated in order to interpret the effectiveness of the CAL package.

## Results

All participants were female and had a mean age of 18.25 ± 2.05 years and 17.81 ± 0.91 years in the CAL package group and the leaflet group, respectively. Regarding the participants' work experience, 87.5% of participants in the CAL package group and 81.3% in the leaflet group had seen 5 or less children with autism. Meanwhile, 6.25% of students in the leaflet group had seen more than 10 children with autism, whereas none of the students in the CAL package group had seen more than 10 children with autism. According to the Shapiro-Wilks W test, the assumptions of normality of retention performance score, level of enjoyment and level of confidence to identify a child with autism were met (W = 0.932, 0.945 and 0.956, respectively). The mean retention performance score of the participants in the CAL package watching group was significantly higher than mean score of the leaflet reading group (87.76% ± 7.68 and 81.25% ± 8.05, p = 0.02, 95% CI = 0.83–12.19, effect size = 0.8). Participants in the CAL package watching group had a higher level of enjoyment than participants in the leaflet reading group (77.25 ± 18.77 and 63.31 ± 18.68, p = 0.04, 95% CI 0.03–2.75, effect size = 0.7). However, there was no statistically significant difference in the level of confidence to identify a child as autism between two groups (Table 1).

**Table 1:** Mean retention performance scores, level of enjoyment and level of confidence for the CAL package and the leaflet groups

	**CAL Package Group (n = 16)**	**Leaflet Group (n = 16)**	**P value**	**95% CI**	Effect size**
**Retention performance score (%, mean ± SD)**	87.76 ± 7.68	81.25 ± 8.05	0.02*	0.83–12.19	0.8

**Level of enjoyment** **(mean ± SD)**	77.25 ± 18.77	63.31 ± 18.68	0.04*	0.03–2.75	0.7

**Level of confidence to identify a child as autism (mean ± SD)**	49.56 ± 19.95	54.94 ± 14.62	0.39	-1.80–0.72	-0.3

* = statistical significance by unpaired Student's t-test

$**\text{Effect size }=\;\frac{\left(\text{mean of the CAL package group }-\text{ mean of the leaflet group}\right)}{\text{pooled standard deviation}}$

When the analysis was carried out of the subcategorised questions, there were no significant differences between the CAL package and leaflet group in the first three subcategories; general knowledge, diagnosis and aetiology. However, the advantage of the CAL package was significantly observed in the last 2 subcategories; co-morbidity (p = 0.02, 95% CI = 1.96–23.03, effect size = 0.9) and treatment (p = 0.003, 95% CI = 4.70–20.30, effect size = 1.2) (Table 2).

**Table 2:** Mean retention performance scores of the subcategorised questions of the CAL package and the leaflet group.

**Questions**	**CAL Package Group (n = 16)**	**Leaflet Group (n = 16)**	**P value**	**95% CI**	Effect size**
**General Knowledge** **(%, mean ± SD)**	67.71 ± 20.61	67.71 ± 12.86	1.0	-12.40–12.40	0.0

**Diagnosis** **(%, mean ± SD)**	90.63 ± 8.54	85.94 ± 8.98	0.14	-1.64–11.02	0.5

**Aetiology** **(%, mean ± SD)**	93.75 ± 25.0	87.5 ± 28.87	0.52	-13.25–25.75	0.2

**Co-morbidities** **(%, mean ± SD)**	95.83 ± 11.38	83.34 ± 17.21	0.02*	1.96–23.03	0.9

**Treatment** **(%, mean ± SD)**	97.5 ± 6.83	85 ± 13.66	0.003*	4.70–20.30	1.2

* = statistical significance by unpaired Student's t-test

$**\text{Effect size }=\;\frac{\left(\text{mean of the CAL package group }-\text{ mean of the leaflet group}\right)}{\text{pooled standard deviation}}$

## Discussion

The findings of this study demonstrated that the childcare students in the CAL package group had a significantly higher retention performance score than those in the leaflet group (p = 0.02, 95% CI = 0.83–12.19, effect size = 0.8). A possible explanation for this finding is that the presentation of more complex information about autism via the visual (images, diagrams, video and animation) and auditory (sounds and narration) channels in the CAL package may increase the efficiency of the participants' working memory. Information entering through both channels simultaneously; for example with the CAL package, can reduce the cognitive load, and resulted in improved learning efficiency [27-29]. Moreover, animations, narration, pictures and sound, if appropriately used in the CAL package, can effectively establish mood, persuasion and explication to the learners [30,31].

Furthermore, students who watched the CAL package performed better in the last two sections of the questionnaire, compared with the leaflet group. This may be due to the ability of the CAL package to hold learners' attention longer compared with reading the leaflet [32]. The use of various presentation styles in the CAL package, e.g. different colours, many text sizes, changing pictures or background, animations and video, may enable learners to maintain their attention better than reading from the printed text [33]. Although, the study found small difference in the retention performance between the CAL package and leaflet groups (p value = 0.02), effect size was relatively high (an effect size of 0.2 is described as "small", 0.5 as "medium" and 0.8 as "large" [34]). However, due to relatively small numbers, caution needs to be taken in interpreting the results. In addition, randomization by a sequence of random numbers may result in selection bias and may lead to imbalance in groups.

In this study, the CAL package group demonstrated a greater level of enjoyment than the leaflet group (p = 0.04, 95% CI = 0.03–2.75, effect size = 0.7). It has face validity that this increased level of enjoyment improved the participants' motivation, which may enhance working memory. In a previous study where level of enjoyment and information recall ability in standard HTML-driven Web sites (predominantly text) users and animated Flash-enhanced sites (integrated animations, moving texts, graphics, rollover effects and sounds; provided the user with a potentially more engaging, immersive, and entertaining experience) users were compared. The Flash presentations were more enjoyable than the text presentations, and the majority preferred using the Flash presentations. In addition, participants who used Flash-enhanced sites were significantly more able to recall information than participants who used HTML sites [35].

The effectiveness of the CAL package was also evaluated by measuring the level of confidence to identify a child as autism. Participants who read the information leaflet had higher confidence in identifying a child with autism than students who watched the CAL package, but the difference did not reach statistical significance (p = 0.39, effect size = -0.3). More than 80% of participants (88% in the CAL package group and 81% in the leaflet group) had seen 5 or less children with autism. However, 6.25% of students in the leaflet group had seen more than 10 children with autism, whereas none of the students in the CAL package group had seen more than 10 children with autism. This finding is a possible explanation of why the CAL package group demonstrated an improved knowledge and enjoyment, but did not show confidence in identifying a child as autism. The findings from the study suggested that, apart from knowledge, experience in working with children with autism is a key factor in determining the level of confidence to identify a child as autism. This is supported by the findings of a recent study in which teachers who had experience with children with autism demonstrated significant greater confidence than teachers who had no or little experience in identifying children as autism [36].

Although, this study suggested a superior effect of the CAL package over the leaflet, there were some limitations, which may affect the validity of the study. Firstly, there was no validity test of the questionnaire. Validation of the questionnaire would have increased the validity of the study. Secondly, in this study, the participants' knowledge about autism prior to interventions was not evaluated. Pre-interventional knowledge would be useful in establishing a baseline level of knowledge of the participants prior to the intervention, thus, an improvement observed in the post-intervention scores could be due to the intervention, rather than due to baseline variations between the groups. Lastly, the study evaluated only enjoyment and acquired knowledge (level 1 and 2 of the Kirkpatrick's hierarchy model), we did not evaluate transfer of knowledge to the workplace and benefits to children with autism as a direct result of the CAL package.

## Conclusion

A computer assisted learning (CAL) package aimed at disseminating knowledge and raise awareness about autism amongst persons who work with children has been developed; and the effectiveness of this CAL package was evaluated, in both educational and entertaining aspects. The study demonstrated that childcare students who received the information via the CAL package had higher retention performance and level of enjoyment compared with students who received the same information via reading the leaflet. These superior factors, however, did not translate to superior confidence in identifying a child as autism. Findings from this study suggest that the CAL package could be used as an alternative teaching material for autism. A study with appropriately powered sample size, validation of assessment tools, and pre-intervention knowledge measurement in the future may be able to confirm findings declared in the study.

## Competing interests

The authors declare that they have no competing interests.

## Authors' contributions

JC designed the study, collected, analysed the data, wrote, and edited the manuscript. BDM and GD designed the study, supervised all the work related to the study and edited the manuscript. All authors read and approved the final manuscript.

## Appendix 1

Examples of the questions in the questionnaire

*Please answer the questions by answering "True or T" if you agree with the sentence or answering "False or F" if you are disagree or "Not sure or NS" if you don't know the answers*.

### General knowledge

.... 1. Autism is an emotional disorder.

.... 2. The symptom varies from normal intelligence to severe disability.

.... 3. Most children with autism have normal intelligence or normal IQ.

### Diagnosis

.... 7. Three main impairments in children with autism are language impairment, social impairment and imagination impairment.

.... 8. Symptom of autism shows since the child is young; therefore, diagnosis can be made at early age.

.... 9. Language impairment is a specific impairment in diagnosing autism because it is found in every child with autism but not be found in other developmental disorders.

### Aetiology

.... 15. The cause of autism is mainly from parenting style.

.... 16. Isolation of the family and the limitations on the family's lifestyle can lead to autism.

### Co-morbidity

.... 17. 70% of children with autism have learning disabilities.

.... 18. Co-existing conditions that should be aware of in children with autism are epilepsy, ADHD, psychiatric disorder such as depression, anxiety, obsessive compulsive disorder, etc.

### Treatment

.... 20. With the proper treatment, the great majority of children with autism are eventually outgrow autism.

.... 21. Treatment of autism requires cooperation with family, carers, teachers and healthcare professionals.

.... 22. Autism can be cure by treatment with medication and diet control.

## Pre-publication history

The pre-publication history for this paper can be accessed here:


